# Association between craniofacial morphological patterns and tooth agenesis-related genes

**DOI:** 10.1186/s40510-020-00309-5

**Published:** 2020-04-06

**Authors:** Amanda Silva Rodrigues, Ellen Cardoso Teixeira, Leonardo Santos Antunes, Paulo Nelson-Filho, Arthur Silva Cunha, Simone Carvalho Levy, Mônica Tirre de Souza Araújo, Alice Gomes de Carvalho Ramos, Giuseppe Valduga Cruz, Marjorie Ayumi Omori, Mírian Aiko Nakane Matsumoto, Alexandre Rezende Vieira, Erika Calvano Küchler, Guido Artemio Marañón-Vásquez, Lívia Azeredo Alves Antunes

**Affiliations:** 1 School of Dentistry, Fluminense Federal University, Rua São Paulo 28, Campus do Valonguinho, Niterói, RJ 24020-150 Brazil; 2 School of Dentistry, Fluminense Federal University, Rua Doutor Sílvio Henrique Braune 22, Niterói, Nova Friburgo, RJ 28625-650 Brazil; 3 Clinical Research Unit, Fluminense Federal University, Rua Mario Santos Braga 30, Niterói, RJ 24020-140 Brazil; 4 Department of Pediatric Dentistry, School of Dentistry of Ribeirão Preto, University of São Paulo, Avenida do Café s/n–Campus da USP, Ribeirão Preto, SP 14040-904 Brazil; 5 Department of Pediatric Dentistry and Orthodontics, School of Dentistry, Federal University of Rio de Janeiro, Rua Prof. Rodolpho Paulo Rocco, 325–Cidade Universitária, Rio de Janeiro, RJ 21941-617 Brazil; 6 Amazonian Education Institute, Rua Maceió 861, Adrianópolis, Manaus, AM 69057-010 Brazil; 7 Department of Oral and Maxillofacial Surgery, Positivo University, Rua Professor Pedro Viriato Parigot de Souza 5300–Campo Comprido, Curitiba, PR 81200-452 Brazil; 8 Department of Oral Biology, School of Dental Medicine, University of Pittsburgh, 412 Salk Pavilion, 335 Sutherland Street, Pittsburgh, PA 15261 USA

**Keywords:** Anodontia, Maxillofacial development, Polymorphism, Genetic

## Abstract

**Background:**

The aim of the present study was to assess if genetic polymorphisms in tooth agenesis (TA)-related genes are associated with craniofacial morphological patterns.

**Methods:**

This cross-sectional, multi-center, genetic study evaluated 594 orthodontic Brazilians patients. The presence or absence of TA was determined by analysis of panoramic radiography. The patients were classified according to their skeletal malocclusion and facial growth pattern by means of digital cephalometric analysis. Genomic DNA was extracted from squamous epithelial cells of buccal mucosa and genetic polymorphisms in *MSX1* (rs1042484), *PAX9* (rs8004560), *TGF-α* (rs2902345), *FGF3* (rs1893047), *FGF10* (rs900379), and *FGF13* (rs12838463, rs5931572, and rs5974804) were genotyped by polymerase chain reaction using TaqMan chemistry and end-point analysis.

**Results:**

Genotypes (*p* = 0.038) and allele (*p* = 0.037) distributions for the *FGF3* rs1893047 were significantly different according to the skeletal malocclusion. Carrying at least one G allele increased in more than two times the chance of presenting skeletal class III malocclusion (OR = 2.21, CI 95% = 1.14–4.32; *p* = 0.017). There was no association between another skeletal craniofacial pattern and some polymorphism assessed in the present study.

**Conclusion:**

Our results suggest that the genetic polymorphism rs1893047 in *FGF3* might contribute to variations in the craniofacial sagittal pattern.

## Background

Tooth agenesis (TA) is the congenital absence of one or more teeth. This condition results from disturbances at early stages of odontogenesis. Several studies have demonstrated that mutations and genetic polymorphisms within specific genes may contribute to the presence of TA; among them are *MSX1* [1]*, PAX9* [1], *TGF-α* [2] and genes from the *FGF* family [3, 4].

*MSX1* (muscle segment homeobox 1) is expressed during epithelial-mesenchymal interactions that occur at the beginning of tooth formation [5]. Mutations on this gene are related to failures in the development of multiple teeth, preferentially premolars [6]. *Msx1* deficient mice showed cleft palate, alveolar bone defects, anomalies in various facial bones, and failure of tooth development [7], suggesting that TA could be genetically related to the development of cranium, maxillary, and mandibular complex [8]. On the other hand, *PAX9* (paired box gene 9) is expressed in the neural-crest-derived mesenchyme of the maxillary/mandibular arches and also contributes to tooth and palate formation [9]. Mutation in *PAX9* gene was associated with autosomal dominant oligodontia, usually affecting the majority of permanent molars [10]. *Pax9* deficient mice presented agenesis of all teeth, cleft palate, and other craniofacial anomalies [5].

*TGF-α* (transforming growth factor-alpha) is a gene expressed during craniofacial development [11]. Although, mice with *Tgf-α* deficiency did not show tooth anomalies [12], human studies evidenced that genetic polymorphisms in *TGF-α* contributes to the presence of TA [2]. Regarding the *FGF* (fibroblast growth factor) signaling, its role in craniofacial development has extensively investigated [13, 14]. This has an inductive function on craniofacial primordia formation and controls the balance among cell growth, differentiation, and apoptosis [13]. *FGF* signaling is expressed during tooth development [15] and it was associated with isolated TA [3, 4].

Some previous studies have reported an association between TA and craniofacial morphological patterns [16–23], including retrognathic maxilla [18], class III skeletal malocclusion [18, 22], and concave profile [21]. Additionally, our recent study demonstrated that TA-associated *GLI2* and *GLI3* genes might play a role in the development of skeletal malocclusions [24]. We therefore reaffirm the hypothesis that TA could share a similar genetic background with specific craniofacial morphologies or skeletal malocclusions, and that genetic polymorphisms on additional TA-associated genes may contribute to the establishment of both conditions. In the present study, we aimed to evaluate whether polymorphisms in TA-related *MSX1*, *PAX9*, *TGF-α*, *FGF3*, *FGF10*, and *FGF13* genes contribute to the development of different craniofacial morphological patterns.

## Materials and methods

The protocol of this study was approved by the Research Ethics Committees of the Antônio Pedro University Hospital at the Fluminense Federal University (n° 33791314.3.0000.5243), School of dentistry of Ribeirão Preto at the University of São Paulo (n° 50765715.3.0000.5419), and the Institutional review board committee at the University of Pittsburgh (no. 12080056). An informed consent form was obtained from all participants or legal guardians.

Orthodontic dental records from 766 ethnically heterogeneous Brazilians were assessed for recruitment (from July 2015 to August 2017). Five hundred and ninety-four orthodontic patients (mean age = 23.1; 238 males, 356 females) from private and graduate orthodontic clinics in Rio de Janeiro (*n* = 325), São Paulo (*n* = 140), and Amazonas cities (*n* = 129) were selected following convenience sampling. Location setting and ethnic composition of each city were already described in previous studies [24, 25]. Patients with one of the following conditions were excluded: previous orthodontic treatment, medical systemic conditions, craniofacial congenital or syndromic anomalies, permanent teeth lost or extracted, and previous facial trauma. Individuals were classified according to the presence/absence of TA, 5.22% presented agenesis of at least one permanent tooth, excluding third molars [24].

### Cephalometric assessment

Pretreatment lateral cephalometric radiographs of patients were scanned using the HP Scanjet G4050 scanner (L1957A; Hewlett Packard, Washington, USA). The images were imported to the Dolphin® Imaging 11.0 software (Dolphin Imaging and Management Solutions, Chatsworth, CA, USA) and then traced and analyzed by calibrated orthodontists. Ten percent of the radiographs were randomly selected and examined twice to test intra- and inter-examiner reproducibility (4-week interval) using the Intraclass Correlation Coefficient (ICC). ICC for repeated measurements ranged from 0.79–0.87.

The ANB angle was used to classify the individuals by their sagittal skeletal malocclusion (class I 0° to 4.0°; class II > 4.0°; class III ANB < 0°). On the other hand, the NaBa-PtGn angle was used to categorize the subjects by their facial growth pattern (mesofacial 87.0°–93.0°, dolichofacial < 87.0°, and brachyfacial > 93.0°).

### DNA extraction and Genotyping

Genomic DNA was extracted from saliva samples for molecular analysis according to a previously reported method [26]. Quantification of the concentration and purity of the DNA was determined using a spectrophotometer (Nanodrop 1000; Thermo Scientific, Wilmington, DE, USA).

Eight genetic polymorphisms, located in intronic regions, were assessed: *MSX1* (rs1042484), *PAX9* (rs8004560), *TGF-α* (rs2902345), *FGF3* (rs1893047), *FGF10* (rs900379), and *FGF13* (rs12838463, rs5931572, and rs5974804) (Table 1). The polymorphisms were blindly genotyped by polymerase chain reactions (PCR) using the TaqMan method (ABI Prism 7900HT, Applied Biosystems, Foster City, CA, USA) [27] and end-point analysis. The interpretation of the data was performed using software provided by Applied Biosystems (Foster City, CA, USA) for allelic discrimination.

**Table 1:** Studied genetic polymorphisms

Gene	Locus	Reference sequence	Type of alteration	Base change (context sequence)	Global MAF
*MSX1*	4p16.2	rs1042484	Intron variant	TCC[A/**G**]ATG	0.1464/733
*PAX9*	14q13.3	rs8004560	Intron variant	TAA[**A**/G]TAT	0.3714/1860
*TGFa*	2p13.3	rs2902345	Intron variant	GGT[C/**T**]GCC	0.4022/2014
*FGF3*	11q13.3	rs1893047	Intron variant	CAC[**A**/G]TGA	0.4545/2276
*FGF10*	5p12	rs900379	Intron variant	CCT[C/**T**]ATA	0.4661/2334
*FGF13*	Xq26.3	rs12838463	Intron variant	ATC[A/**G**]TAG	0.4437/1675
*FGF13*	Xq27.1	rs5931572	Intron variant	ATT[A/**G**]TTT	0.4575/1727
*FGF13*	Xq27.1	rs5974804	Intron variant	CAT[C/**T**]GGT	0.4967/1875

Source of information: dbSNP from: https://www.ncbi.nlh.nih.gov/snp/, http://genome.uscs.edu/, and https://www.thermofisher.com

Bold: lower frequency allele

### Statistical analysis

Statistical analyses were performed on Epi Info 3.5.2 (www.cdc.gov/epiinfo) and Plink (http://zzz.bwh.harvard.edu/plink/), using an established *α* of 0.05. Chi-square test (with Yates’ correction for continuity, when necessary) or Fisher’s exact test were performed to determine association between allele/genotype frequencies and the craniofacial phenotypes assessed. For the polymorphisms located in the chromosome X (polymorphisms in *FGF13*), an analysis adjusted by the gender was performed. Due to the multiple comparisons made, a Bonferroni correction was applied for each evaluated outcome (corrected *p* value = 0.00625; 0.05/8 genetic polymorphisms assessed). Genotype/phenotype associations were also tested in the dominant and recessive models. Chi-square test was also used to evaluate the Hardy-Weinberg equilibrium.

## Results

The distribution of genotypes followed Hardy-Weinberg equilibrium (data not shown). Information regarding the association between skeletal malocclusion and TA was reported in a previous published paper [24]; individuals presenting class II skeletal malocclusion showed lower frequency of TA. There was no significant association between genotype/allele distributions and the presence of TA for any polymorphism assessed in the present study (*p* > 0.05) (Table 2).

**Table 2:** Genotype and allele distribution between TA and non-TA participants

Genetic polymorphism	Genotypes, *n* (%)			*p* value	Alleles, *n* (%)		*p* value
***MSX1*rs1042484**	**AA**	**AG**	**GG**		**A**	**G**	
**TA**	12 (66.7)	3 (16.7)	3 (16.7)	0.414	27 (75.0)	9 (25.0)	0.475
**Non-TA**	239 (70.5)	73 (21.5)	27 (8.0)		551 (81.3)	127 (18.7)	
***PAX9*rs8004560**	**AA**	**AG**	**GG**		**A**	**G**	
**TA**	11 (57.9)	6 (31.6)	2 (10.5)	0.783	28 (73.7)	10 (26.3)	0.740
**Non-TA**	214 (65)	81 (24.6)	34 (10.3)		509 (77.4)	149 (22.6)	
***TGFα1*rs2902345**	**CC**	**CT**	**TT**		**C**	**T**	
**TA**	6 (23.1)	12 (46.2)	8 (30.8)	0.495	24 (46.2)	28 (53.8)	0.317
**Non-TA**	111 (30.0)	180 (48.6)	79 (21.4)		402 (54.3)	338 (45.7)	
***FGF3*rs1893047**	**AA**	**AG**	**GG**		**A**	**G**	
**TA**	17 (68)	7 (28)	1 (4)	0.507	41 (82.0)	9 (18.0)	0.881
**Non-TA**	359 (74.4)	92 (19)	32 (6.6)		810 (83.8)	156 (16.2)	
***FGF10*rs900379**	**CC**	**CT**	**TT**		**C**	**T**	
**TA**	10 (35.7)	11 (39.3)	7 (25)	0.775	24 (46.2)	28 (53.8)	0.555
**Non-TA**	164 (29.4)	244 (43.8)	149 (26.8)		572 (51.3)	542 (48.7)	
***FGF13*rs12838463**	**AA**	**AG**	**GG**		**A**	**G**	
**TA**	19 (51.4)	9 (24.3)	9 (24.3)	0.828	47 (63.5)	27 (36.5)	0.306
**Non-TA**	360 (46.2)	212 (27.2)	207 (26.6)		932 (59.8)	626 (40.2)	
***FGF13*rs5931572**	**AA**	**AG**	**GG**		**A**	**G**	
**TA**	9 (32.1)	10 (35.8)	9 (32.1)	0.783	28 (50.0)	28 (50.0)	0.340
**Non-TA**	170 (32.1)	160 (30.2)	200 (37.7)		500 (47.2)	560 (52.8)	
***FGF13*rs5974804**	**AA**	**AG**	**GG**		**A**	**G**	
**TA**	8 (40)	6 (30)	6 (30)	0.884	22 (55.0)	18 (45.0)	0.520
**Non-TA**	194 (43.5)	112 (25.10)	140 (31.4)		500 (56.1)	392 (43.9)	

*Statistical significance (*p* ≤ 0.05). For genetic polymorphisms in *FGF13*, analyses were adjusted by the gender

Genotype (*p* = 0.038) and allele (*p* = 0.037) distributions for the *FGF3* rs1893047 were significantly different between class III and class I individuals (Table 3). Analysis in the dominant model (AG + GG vs. AA) demonstrated that carrying at least one G allele increased in more than two times the chance of presenting skeletal class III malocclusion (OR = 2.21, 95% CI = 1.14–4.32; *p* = 0.017). There was no association between the facial growth pattern and any polymorphism assessed in the present study (*p* > 0.05) (Table 4). No reported associations remained significant after the Bonferroni correction.

**Table 3:** Genotype and allele distribution among class I, class II and class III skeletal malocclusions

Genetic polymorphism	Genotypes, *n* (%)			*p* value	Alleles, *n* (%)		*p* value
***MSX1*rs1042484**	**AA**	**AG**	**GG**		**A**	**G**	
**Class I**	118 (69.8)	38 (22.5)	13 (7.7)	Reference	274 (81.1)	64 (18.9)	Reference
**Class II**	106 (71.1)	29 (19.5)	14 (9.4)	0.730	241 (80.9)	57 (19.1)	0.999
**Class III**	26 (70.3)	8 (21.6)	3 (8.1)	0.990	60 (81.1)	14 (18.9)	0.863
***PAX9*rs8004560**	**AA**	**AG**	**GG**		**A**	**G**	
**Class I**	91 (62.3)	35 (24.0)	20 (13.7)	Reference	217 (74.3)	75 (25.7)	Reference
**Class II**	100 (67.1)	39 (26.2)	10 (6.7)	0.140	239 (80.2)	59 (19.8)	0.108
**Class III**	25 (62.5)	10 (25.0)	5 (12.5)	0.975	60 (75.0)	20 (25.0)	0.999
***TGFα1*rs2902345**	**CC**	**CT**	**TT**		**C**	**T**	
**Class I**	51 (28.5)	89 (49.7)	39 (21.8)	Reference	191 (53.4)	167 (46.6)	Reference
**Class II**	46 (28.8)	77 (48.1)	37 (23.1)	0.947	169 (52.8)	151 (47.2)	0.999
**Class III**	16 (37.2)	22 (51.2)	5 (11.6)	0.261	54 (62.8)	32 (37.2)	0.144
***FGF3*rs1893047**	**AA**	**AG**	**GG**		**A**	**G**	
**Class I**	157 (76.6)	36 (17.6)	12 (5.9)	Reference	350 (85.4)	60 (14.6)	Reference
**Class II**	165 (75.3)	39 (17.8)	15 (6.8)	0.932	369 (84.2)	69 (15.8)	0.964
**Class III**	28 (59.6)	16 (34.0)	3 (6.4)	0.038*	72 (76.6)	22 (23.4)	0.037*
***FGF10*rs900379**	**CC**	**CT**	**TT**		**C**	**T**	
**Class I**	70 (29.8)	107 (45.5)	58 (24.7)	Reference	247 (52.6)	223 (47.4)	Reference
**Class II**	72 (30.5)	115 (48.7)	49 (20.8)	0.585	259 (54.9)	213 (45.1)	0.509
**Class III**	19 (33.3)	20 (35.1)	18 (31.6)	0.336	58 (50.9)	56 (49.1)	0.103
***FGF13*rs12838463**	**AA**	**AG**	**GG**		**A**	**G**	
**Class I**	110 (47.8)	65 (28.3)	55 (23.9)	Reference	285 (62.0)	175 (38.0)	Reference
**Class II**	112 (46.9)	60 (25.1)	67 (28.0)	0.541	284 (59.4)	194 (40.6)	0.765
**Class III**	29 (50.9)	14 (24.6)	14 (24.6)	0.850	72 (63.2)	42 (36.8)	0.876
***FGF13*rs5931572**	**AA**	**AG**	**GG**		**A**	**G**	
**Class I**	72 (32.6)	63 (28.5)	86 (38.9)	Reference	207 (46.8)	235 (53.2)	Reference
**Class II**	67 (29.5)	65 (28.6)	95 (41.9)	0.749	199 (43.8)	255 (56.2)	0.823
**Class III**	16 (30.8)	15 (28.8)	21 (40.4)	0.966	47 (45.2)	57 (54.8)	0.091
***FGF13*rs5974804**	**AA**	**AG**	**GG**		**A**	**G**	
**Class I**	83 (42.1)	50 (25.4)	64 (32.5)	Reference	216 (54.8)	178 (45.2)	Reference
**Class II**	80 (44.7)	48 (26.8)	51 (28.5)	0.897	208 (58.1)	150 (41.9)	0.820
**Class III**	22 (46.8)	9 (19.1)	16 (34.0)	0.678	53 (56.4)	41 (43.6)	0.784

*Statistical significance (*p* ≤ 0.05). For genetic polymorphisms in *FGF13*, analyses were adjusted by the gender.

**Table 4:** Genotype and allele distribution among mesofacial, dolichofacial, and brachyfacial growth patterns

Genetic polymorphism	Genotypes, *n* (%)			*p* value	Alleles, *n* (%)		*p* value
***MSX1*rs1042484**	**AA**	**AG**	**GG**		**A**	**A**	
**Mesofacial**	119 (69.6)	35 (20.5)	17 (9.9)	Reference	273 (79.8)	69 (20.2)	Reference
**Dolichofacial**	77 (70.6)	22 (20.2)	10 (9.2)	0.975	176 (80.7)	42 (19.3)	0.888
**Brachyfacial**	54 (72.0)	18 (24.0)	3 (4.0)	0.271	126 (84)	24 (16)	0.335
***PAX9*rs8004560**	**AA**	**AG**	**GG**		**A**	**G**	
**Mesofacial**	103 (66.5)	37 (23.9)	15 (9.7)	Reference	243 (78.4)	67 (21.6)	Reference
**Dolichofacial**	70 (63.1)	28 (25.2)	13 (11.7)	0.811	168 (75.7)	54 (24.3)	0.527
**Brachyfacial**	43 (62.3)	19 (27.5)	7 (10.1)	0.823	105 (76.1)	33 (23.9)	0.680
***TGFα1*rs2902345**	**CC**	**CT**	**TT**		**C**	**T**	
**Mesofacial**	51 (27.4)	95 (51.1)	40 (21.5)	Reference	197 (53.0)	175 (47)	Reference
**Dolichofacial**	35 (30.2)	55 (47.4)	26 (22.4)	0.815	125 (53.9)	107 (46.1)	0.888
**Brachyfacial**	27 (33.8)	38 (47.5)	15 (18.8)	0.574	92 (57.5)	68 (42.5)	0.383
***FGF3*rs1893047**	**AA**	**AG**	**GG**		**A**	**G**	
**Mesofacial**	166 (75.1)	39 (17.6)	16 (7.2)	Reference	371 (83.9)	71 (16.1)	Reference
**Dolichofacial**	111 (75.5)	28 (19.0)	8 (5.4)	0.765	250 (85.0)	44 (15.0)	0.865
**Brachyfacial**	73 (70.9)	24 (23.3)	6 (5.8)	0.464	170 (82.5)	36 (17.5)	0.886
***FGF10*rs900379**	**CC**	**CT**	**TT**		**C**	**T**	
**Mesofacial**	78 (31.0)	118 (46.8)	56 (22.2)	Reference	274 (54.4)	230 (45.6)	Reference
**Dolichofacial**	49 (30.2)	73 (45.1)	40 (24.7)	0.873	171 (52.8)	153 (47.2)	0.871
**Brachyfacial**	34 (29.8)	51 (44.7)	29 (25.4)	0.658	119 (52.2)	109 (47.8)	0.798
***FGF13*rs12838463**							
**Mesofacial**	116 (47.0)	63 (25.5)	68 (27.5)	Reference	295 (59.7)	199 (40.3)	Reference
**Dolichofacial**	79 (47.6)	43 (25.9)	44 (26.5)	0.911	201 (60.5)	131 (39.5)	0.981
**Brachyfacial**	56 (49.6)	33 (29.2)	24 (21.2)	0.624	145 (64.2)	81 (35.8)	0.678
***FGF13*rs5931572**	**AA**	**AG**	**GG**		**A**	**G**	
**Mesofacial**	70 (29.2)	69 (28.8)	101 (42.1)	Reference	209 (43.5)	271 (56.5)	Reference
**Dolichofacial**	54 (35.3)	40 (26.1)	59 (38.6)	0.443	148 (48.4)	158 (51.6)	0.746
**Brachyfacial**	31 (29.0)	34 (31.8)	42 (39.3)	0.830	96 (44.9)	118 (55.1)	0.104
***FGF13*rs5974804**	**AA**	**AG**	**GG**		**A**	**G**	
**Mesofacial**	84 (42.6)	49 (24.9)	64 (32.5)	Reference	217 (55.1)	177 (44.9)	Reference
**Dolichofacial**	60 (46.9)	33 (25.8)	35 (27.3)	0.602	153 (59.8)	103 (40.2)	0.139
**Brachyfacial**	41 (41.8)	25 (25.5)	32 (32.7)	0.989	107 (54.6)	89 (45.4)	0.912

*Statistical significance (*p* ≤ 0.05). For genetic polymorphisms in *FGF13*, analyses were adjusted by the gender.

## Discussion

Many human and animal studies support that dental anomalies, mainly TA, and craniofacial alterations could share a common genetic background [7, 9, 12–24, 28, 29]. The identification of genes contributing to the establishment of craniofacial morphological patterns can impact the clinical practice, allowing genetic counseling of individuals carrying specific variants and their families, and to work on preventive strategies. To the best of our knowledge, this is the first report investigating the association between genetic polymorphisms in TA-related genes—*MSX1*, *PAX9*, *TGF-α*, and *FGF* signaling–and craniofacial morphological patterns (associated or not with TA).

*MSX1*, *PAX9*, *TGF-α*, and *FGF* genes are responsible for the patterning of tooth development [7, 9, 15, 30]. Previous studies performed in Brazilian families (trio designs) and case-controls studies demonstrated that TA was associated with *MSX1*, *PAX9*, *TGF-α* [2]*, FGF3* [2], and *FGF10* [3]*.* In the present study, none of the studied genetic polymorphisms were associated with TA. These results should be carefully evaluated, because they may be due to methodological limitations of the present study (small sample size of individuals with TA, sampling limitations, failure rate of genotyping procedures), which could have led to type II error.

On the other hand, it has been demonstrated that *Msx1* deficient mice show shortened mandibles, anteroposterior deficiency in the middle third of the face, and subtle abnormalities in overall head size and cranial shape [7]. Also, *Pax9* deficient mice present agenesis of all teeth, cleft palate, and other craniofacial anomalies [5]. *TGF-α* is a gene expressed during craniofacial development [11]; mice with *Tgf-α* deficiency presented eye and hair anomalies [12]. Despite the above-described roles of these genes, the genetic polymorphisms studied within *MSX1*, *PAX9*, and *TGF-α* were not associated with none of the craniofacial morphological patterns assessed (skeletal malocclusions and facial type). Considering that previous studies support that genetic polymorphisms and mutations in these genes did show association with craniofacial patterns [2, 6, 10, 31–33], it is possible that other genetic variants within these genes are involved in the etiology of the craniofacial phenotypes in this population.

Fgf signaling is involved in various regulatory processes during embryogenesis as well as in the adult organism [34, 35]. This signal pathway has key roles in suture and synchondrosis regulation; mutations in *FGF* receptors cause craniosynostosis, which is the premature suture fusion [36, 37]. On the other hand, Fgf signal participates in multiple stages of palatogenesis, from palatal shelf elevation to the completion of fusion [38]. Therefore, disturbances in Fgf-related pathways are possible mechanisms of palatal cleft. Based on the above, it is clear that *FGF* is considered a candidate gene for study in relation to variations in the morphology of the craniofacial skeleton. In our study, carrying at least one G allele in the polymorphism rs1893047 within *FGF3* increased the chance of presenting skeletal class III malocclusion. Considering that multiple members of this gene family are expressed mainly during midfacial region development [39], among them *FGF3*, we hypothesize that variations on this gene could contribute to the development of class III due to maxillary growth deficiency. A stratified analysis according to the maxilla contribution on class III was not possible due to the low prevalence of class III individuals in our sample, and the consequent significant reduction that would have existed in the power of the analyses.

It has been shown that *Fgf3* is expressed during development and outgrowth of the facial primordia and branchial arches [39–41], and during odontogenesis [42, 43]. Additionally, this gene has been associated with oral cleft [31] and TA [2], suggesting that *FGF3* plays a role in both craniofacial phenotypes. Our data must be carefully interpreted since significant results could be due to chance. Statistical significance did not persist for the reported associations after the Bonferroni correction.

Briefly, the knowledge regarding the role of genetic polymorphisms on craniofacial development offers the possibility of establishing new strategies to prevent these disorders. Further investigations with other genetic polymorphisms in these genes are necessary to confirm our results.

## Conclusion

Our result suggests that genetic polymorphism rs1893047 in *FGF3* might contribute to variations in the craniofacial sagittal pattern, specifically to the establishment of the Class III skeletal malocclusion.

## Data Availability

The data will be made available upon request to the authors.

## Abbreviations

TA: Tooth agenesis
ICC: Intraclass correlation coefficient
PCR: Polymerase chain reaction
